# High-throughput capturing and characterization of mutations in essential genes of *Caenorhabditis elegans*

**DOI:** 10.1186/1471-2164-15-361

**Published:** 2014-05-12

**Authors:** Jeffrey Shih-Chieh Chu, Shu-Yi Chua, Kathy Wong, Ann Marie Davison, Robert Johnsen, David L Baillie, Ann M Rose

**Affiliations:** Department of Medical Genetics, University of British Columbia, Vancouver, Canada; Department of Molecular Biology and Biochemistry, Simon Fraser University, Burnaby, Canada; Department of Biology, Kwantlen Polytechnic University, Surrey, Canada

**Keywords:** Whole genome sequencing, EMS, Mutagenesis, Essential genes, Balanced mutation, Lethal mutation, *C. elegans*, Cell cycle

## Abstract

**Background:**

Essential genes are critical for the development of all organisms and are associated with many human diseases. These genes have been a difficult category to study prior to the availability of balanced lethal strains. Despite the power of targeted mutagenesis, there are limitations in identifying mutations in essential genes. In this paper, we describe the identification of coding regions for essential genes mutated using forward genetic screens in *Caenorhabditis elegans*. The lethal mutations described here were isolated and maintained by a wild-type allele on a rescuing duplication.

**Results:**

We applied whole genome sequencing to identify the causative molecular lesion resulting in lethality in existing *C. elegans* mutant strains. These strains are balanced and can be easily maintained for subsequent characterization. Our method can be effectively used to analyze mutations in a large number of essential genes. We describe here the identification of 64 essential genes in a region of chromosome I covered by the duplication *sDp2.* Of these, 42 are nonsense mutations, six are splice signal mutations, one deletion, and 15 are non-synonymous mutations. Many of the essential genes in this region function in cell cycle, transcriptional regulation, and RNA processing.

**Conclusions:**

The essential genes identified here are represented by mutant strains, many of which have more than one mutant allele. The genetic resource can be utilized to further our understanding of essential gene function and will be applicable to the study of *C. elegans* development, conserved cellular function, and ultimately lead to improved human health.

**Electronic supplementary material:**

The online version of this article (doi:10.1186/1471-2164-15-361) contains supplementary material, which is available to authorized users.

## Background

The proper development and viability of an organism is dependent on a group of genes called essential genes. In humans, gene essentiality has been long associated with many diseases such as miscarriages [1, 2], heritable diseases, and cancer [3]. Recent studies have shown that over-expression of some essential genes promotes cell proliferation in cancer [4]. Due to its importance for survival, essential genes have been targets for new therapeutics or antimicrobials [5]. To effectively study essential genes, generating lethal alleles in model systems is required. In the nematode *Caenorhabditis elegans*, the essential gene set is the largest set of genes and is estimated to contain 25% of all the genes [6–9]. Using RNAi, about 3500 genes have been annotated as essential (data collected from WormBase [10, 11]). Inparanoid, a sequence based orthology inference tool, detects about 40% of the *C. elegans* genes are orthologous to the human genes. But approximately 60% of the essential genes show clear human orthologs, showing high conservation of essential genes, which makes *C. elegans* an excellent platform for examination of essential gene functions that are relevant to human health. Many important genes, such as *let-60*/Ras [12] and *let-740/dcr-1*[13, 14], were first discovered through *C. elegans* genetics. However, the genetic resource for studying these genes is severely lacking. Even with the concerted community effort such as the *C. elegans* Deletion Mutant Consortium [15], mutations in many essential genes are still lacking in the knock-out collection. The consortium have generated close to 6000 knock-out strains since 1998, but only 1436 essential genes are in the current collection [16, 17]. In addition to the considerable time and effort required to generate a single knock-out allele, an outstanding disadvantage of the targeted deletion approach is that extra effort is needed to balance the lethal mutation [18]. Recently, the Consortium has adopted a procedure of random mutagenesis followed by whole genome sequencing (WGS) to generate and identify a large number of mutations [15]. Although this project can generate more mutations in shorter time, their method does not capture mutations that exhibit lethal phenotypes, and thus, essential genes are selected against. This outcome indicates thousands of essential genes do not have knockout alleles.

To complement the effort of the *C. elegans* community, we took advantage of the balancer system, which was developed 30 years ago for capturing and maintaining lethal mutations, with the next-generation DNA sequencing technologies. Almost 70% of the *C. elegans* genome have been successfully balanced by large genomic rearrangements [18]. By mutagenizing a pre-balanced strain removes the need to perform additional genetic crosses to balance a lethal mutation. The balancer system, designed specifically to capture and maintain lethal mutations, is the system of choice for generating mutations in essential genes. Such screens have been carried out for regions in chromosome I [19, 20], II [21], III [22], IV [23–26], V [27], and X [28, 29]. In our laboratories, we have generated over 1350 lethal mutations that fall into 486 complementation groups.

The next hurdle in the analysis of essential genes is the molecular identification of the genomic lesion, which to date has involved an enormous effort. Traditional methods of gene cloning that rely upon candidate identification of mapped mutations can take months or years. This gene-by-gene approach was only able to characterize 30 essential genes from our library to date. This problem has been difficult to solve until the recent advances in sequencing technology. To address the problem of coding region identification, we have recently developed a fast and scalable pipeline that takes advantage of whole genome sequencing and bioinformatics analysis to identify the causal mutation responsible for the lethal phenotype [30]. Recent studies, including our initial analysis of *let-504*[30], have shown that whole genome sequencing is an efficient and cost-effective approach to identifying the encoded gene product especially when there are additional alleles that can be sequenced to provide confirmation [30–34]. In this paper, we describe our approach of combining an established mutagenesis technique with the latest sequencing technology in order to close the gap in the essential gene knock-out collection.

## Results and discussion

### Chromosome I left has a high percentage of essential genes

The leftmost 7.3 Mbp of chromosome I has the highest percentage of mapped essential genes and closest to saturation with 237 essential genes isolated and mapped [19]. The mutant strains were derived by mutagenizing KR235 [*dpy-5 (e61)*, +, *unc-13 (e450)*/*dpy-5(e61)*, *unc-15(e73)*, +; *sDp2*] with a low dose of EMS and isolating *let-x dpy-5 unc-13* homozygotes rescued with a third wild-type allele of *dpy-5* and *let-x* balanced by free duplication *sDp2*[35] (see schematic in Additional file 1)*.* In order to position the genes, mutations were mapped into 60 zones using a combination of three-factor mapping and complementation to a series of duplications and deficiencies [19]. Within zones, lethal mutants were inter-complementation tested. The earliest developmental arrest stages were determined for each complementation group [19]. The candidate lesions are present in two copies and rescued by a third wild-type allele on *sDp2.* Thus, our high throughput identification method focused on finding heterozygous mutations that exhibit an allelic ratio between the range of 40% to 90% [30]. In order to assess the accuracy of our recently developed high throughput method [30], we selected 81 genes from this set with the criteria that they formed a complementation group having more than one allele (Additional file 2). The extra alleles provide an added resource for validation. We sequenced 10 indexed genomic DNA samples per Illumina HiSeq lane and obtained a total of 385 Gbp of sequence. The sequencing reads were aligned using BWA [36] to the WS200 *C. elegans* reference sequence. We achieved 30X coverage on average across the whole genome and an average of 35X coverage in coding elements. In the case of two strains, only 6X coverage was obtained: *let-369(h125)* and *let-594(h407)*. Genomes from these two strains were removed from subsequent analysis.

### The mutational landscape provided a quality check

Our first analytical step, as a quality check, was to confirm the presence of the *dpy-5* (*e61)* and *unc-13* (*e450)* mutations in each genome. For *unc-13*, the expected variant ratio should be 100% because the duplication does not extend far enough to provide an additional wild-type allele. For *dpy-5* however, there is a wild-type allele on *sDp2*, and thus we would expect to see a 66% variant ratio. We found the expected ratios in 76 of the 79 genomes. Three genomes deviated from the norm: *let-516(h144)* is missing both *e450* and *e61* (all the reads supported the reference sequence); *let-388(h88)* is missing *e61*; *let-393(h225)* has *e61*, but with a 33% ratio rather than the expected 66%. We examined these strains for the presence of the duplication *sDp2*. When the duplication is present, the read depth is 33% greater in the first 7Mbp of chromosome I than for rest of the chromosome. Our analysis showed that none of these three genomes showed any depth difference (Additional file 3). It is likely these strains do not carry *sDp2*. Although *sDp2* does not crossover with the normal homologs at a readily detectable frequency, rare exchange events can occur resulting in subsequent loss of the duplicated fragment [37]. These three strains were not analyzed further.

### Coding sequence correlated with high confidence

We analyzed the parental strain KR235 and identified 571 SNVs and 167 small indels that show >40% read support on Chromosome I when compared to the *C. elegans* WS200 reference using VarScan (see Methods). These mutations represent the background mutations in which the lethal mutations were maintained. For the remaining 76 genomes, we filtered out the background mutations and found on average 44 SNVs that show >40% allelic ratio in the *sDp2* region. Most of the SNVs are G > A or C > T changes as expected and previously observed after EMS treatment [30, 38]. We also found an average of 7 small indels of 1–2 bps. We categorized each mutation as either nonsense, missense, synonymous, splice signal disruption, frame shifting indel, frame preserving indel, or noncoding mutation. Noncoding mutations were defined as any mutation located outside of coding regions. A full list of SNVs and indels, for each strain, is available on our server at http://lethal.mbb.sfu.ca/jschu/essential_genes.

We identified candidate mutations for the 76 genomes using our bioinformatics pipeline that we developed previously [30] (see also Methods) and validated a subset of our candidates by sequencing a second allele or by complementation testing (Table 1). Nine of our candidate lesions were in genes that had been previously identified and published. In a few cases, candidates expected to be in separate genes were located in the same coding region. These observations were confirmed by further genetic complementation tests (Additional file 4). Previously identified *let-631* and *let-103* were found to be allelic to *let-363.* As a result, *let-363* gains three new *sDp2*-balanced alleles (*h216, h451, h502*) in addition to the nine existing ones. *let-519* and *let-104* are allelic to *let-526* and thus *let-526* gains four new alleles: *h799*, *h373*, *h405* and *h526. let-630* fails to complement *let-596* and now has five alleles: *h355*, *h702*, *h432*, *h782*, and *h258*. Thirty-five candidates were tested by sequencing a second allele using previously published complementation data [19]. Of these, we confirmed 29 identities. All in all, we tested 48 candidates and confirmed 42 (87.5%). For the remaining 28 genomes, we have high confidence in the identity of 22 genes based on their map position. Thus, including previously described *let-504*, we now have coding region assignments for 64 *let-* genes in the *sDp2* region. Because the genes in this study all have multiple alleles, thus by inference, we have confidently identified the coding regions affected in a total of 259 mutant strains (Additional file 2).

**Table 1:** **Coding DNA Sequence (CDS) identifications of** ***let-*** **genes**

Gene	Allele	Allele mutation	Molecular identity	Support	Confirmation status	Human ortholog	Associated human conditions	References
lin-6/mcm-4	h92	C > *	Mini chromosome maintenance	RNAi	Confirmed^1^	MCM4	Natural killer cell and glucocorticoid deficiency with DNA repair defect	[39]
let-354/dhc-1	h79	Q > *	Dynein heavy chain	Both	Confirmed^1^	DYNC1H1	Charcot-Marie-Tooth disease, Mental retardation, Spinal muscular atrophy	[40–42]
let-502/rock	h392	Q > *	Rho associated kinase	RNAi	Confirmed^1^	ROCK1		[43]
let-363/tor	h98	Splice variant	Tor kinase	Both	Confirmed^1^	MTOR	pancreatic neuroendocrine tumors	[44, 45]
	h420^a^	Q > *			Confirmed^3^			
	h502^a^	Splice variant			Confirmed^3^			
let-603/air-2	h289	W > *	Aurora-related serine/threonine kinase	Both	Confirmed^1^	AURKA	Susceptibility to colon cancer	[46]
let-512/vps-34	h797	P > S	phosphoinositide 3-kinase		Confirmed^1^	PIK3C3		[47]
let-381/foxf	h107	splice variant	Forkhead transcription factor F	K.O.	Confirmed^1^	FOXF2		[48]
let-607/bZip	h402	Q > *	Leucine zipper transcription factor	Both	Confirmed^1^	CREB3L3		[49]
let-504/E01A2.4	h448	M > I	NFkB activating protein	Both	Confirmed^1^	NKAP		
let-152/ccb-1	h685	W > *	Calcium channel subunit		Confirmed^2^	CACNB2	Brugada syndrome 4	
let-355/hel/T05E8.3	h81	Y > *	DEAD/H helicase	RNAi	Confirmed^2^	DHX33		
let-362/rhel/Y71G12B.8	h86	R > *	DEAD/H RNA helicase	RNAi	Confirmed^2^	DDX27		
let-366/aars-2	h112	Q > *	Alanine tRNA synthetase	RNAi	Confirmed^2^	AARS	Charcot-Marie-Tooth disease	[50]
let-368/inx-12	h121	W > *	Innexin gap junction	K.O.	Confirmed^2^			
let-370/coq-1	h128	G > E	hexaprenyl pyrophosphate synthetase	K.O.	Confirmed^2^	PDSS1	Coenzyme Q10 deficiency, Parkinson’s disease	[51]
let-389/nars-1	h680	G > E	Asparagine tRNA synthetase	Both	Confirmed^2^	NARS		
let-396/fars-1	h217	Q > *	Phenylalanine tRNA synthetase	RNAi	Confirmed^2^	FARSA		
let-522/hlh-2	h735	W > *	Helix loop helix transcription factor	Both	Confirmed^2^	TCF3	Acute lymphoblastic leukemia	
let-529/asd-2	h238	Q > *	KH domain containing RNA binding protein	RNAi	Confirmed^2^	QKI	Mental retardation	
let-575/ptr-2	h345	W > *	Sterol sensing domain protein	RNAi	Confirmed^2^	PTCHD1	Autism spectrum disorders	[52–54]
let-585/inx-13	h784	W > *	Innexin gap junction	RNAi	Confirmed^2^			
let-595/imb-1	h353	R > *	Importin	RNAi	Confirmed^2^	KPNB1		
let-598/F27C1.6	h213	Q > *	U3 small nucleolar ribonucleoprotein	RNAi	Confirmed^2^	UTP14C		
let-599/nath-10	h290	L > F	N-acetyl transferase	Both	Confirmed^2^	NAT10		
let-608/ncbp-1	h706	Q > *	Nuclear cap binding protein	RNAi	Confirmed^2^	NCBP1		
let-611/C48E7.2	h756	Q > *	RNA polymerase III subunit	RNAi	Confirmed^2^	POLR3C		
let-612/apm-1	h466	splice variant	Adaptin subunit	RNAi	Confirmed^2^	AP1M1		
let-365/sep-1	h108	W > *	Separase	Both	Confirmed^2^	ESPL1	Breast cancer oncogene	
let-364/mat-1	h104	S > F	Anaphase promoting complex subunit	RNAi	Confirmed^2^	CDC27		
let-101/npp-6	h242	W > *	Nuclear pore complex protein	Both	Confirmed^2^	NUP160		
let-106/hcp-6	h787	C > Y	Condensin subunit	Both	Confirmed^2^	NCAPD3		
let-379/tag-345	h127	W > *	Nucleolar protein complex member	RNAi	Confirmed^2^	WDR12		
let-503/R12E2.2	h313	Q > *	Protein of unknown function	K.O.	Confirmed^2^	SUCO		
let-517/spg-7	h264	G > E	Metalloprotease	Both	Confirmed^2^	AFG3L2	Spastic ataxia, Spinocerebellar ataxia	
let-597/hcp-4	h349	E > *	Holocentromeric protein	RNAi	Confirmed^2^	CENPC		
let-630/Y110A7A.19	h355^b^	R > *	Pentatricopeptide repeat containing protein	RNAi	Confirmed^2^	PTCD3		
	h782^b^	W > *			Confirmed^2^			
let-646/pat-10	h233	G > E	Troponin C	RNAi	Confirmed^2^	TNNC1	Cardiomyopathy	
let-526	h799^c^	Q > *	SWI/SNF complex subunit	Both	Confirmed^3^	ARID1A	Mental retardation	
	h405^c^	W > *			Confirmed^3^			
let-129/zfh-2	h379	Q > *	zinc finger homeobox protein	Both	Prediction	ZFHX3, ZFHX4	Susceptibility to prostate cancer, Ptosis	
let-147/rnp-6	h463	G > E	RNA splicing factor	RNAi range	Prediction	PUF60	Verheij syndrome	
let-373/unc-73	h234	Del	Guanine nucleotide exchange factor	Both	Prediction	TRIO		
let-377/lim-7	h110	W > *	LIM homeodomain protein	K.O.	Prediction	ISL2		
let-378/dnj-21	h124	G > E	DnaJ domain containing protein	RNAi	Prediction	DNAJC15		
let-380/knl-2	h80	W > *	Centromeric protein	Both	Prediction			
let-382/nuo-2	h82	Q > *	Mitochondria complex I subunit	Both	Prediction	NDUFS3	Leigh syndrome, Mitochondrial complex I deficiency	
let-383/T21G5.6	h115	W > *	Protein of unknown function		Prediction			
let-384/C06A5.1	h84	Q > *	Integrator subunit	RNAi	Prediction	INTS1		
let-385/teg-4	h85	splice variant	splicing factor	RNAi	Prediction	SF3B3		
let-386/dbr-1	h117	G > E	RNA lariat-debranching enzyme	RNAi range	Prediction	DBR1		
let-391/tag-146	h91	Q > *	Uncharacterized zinc finger protein	K.O.	Prediction			
let-397/rpb-5	h228	Q > *	RNA polymerase II subunit	RNAi	Prediction	POLR2E		
let-400/prpf-4	h269	D > G	Pre-mRNA processing factor	RNAi	Prediction	PRPF4B		[55]
let-509/unc-73	h142	W > *	Guanine nucleotide exchange factor	Both	Prediction	TRIO		
let-527/nhr-23	h207	R > Q	Nuclear hormone receptor	Both	Prediction	RORC		
let-534/ahcy-1	h260	Q > *	S-adenosylhomocysteine hydrolase	Both	Prediction	AHCY	Hypermethioninemia	
let-581/unc-11	h725	A > V	clathrin adaptor protein	RNAi; Range	Prediction	PICALM	Acute lymphoblastic leukemia, Acute T-cell lymphoblastic leukemia	
let-601/cuti-1	h281	Q > *	Cuticle regulatory protein	Both	Prediction			
let-602/T09B4.9	h283	W > *	translocase	RNAi	Prediction	TIMM44		
let-604/mdt-18	h293	splice variant	Mediator subunit	RNAi	Prediction	MED18		
let-605/cye-1	h312	W > *	E-type cyclin	Both	Prediction	CCNE1		
let-614	h138				Tested against F27C1.3 but did not confirm			
let-376	h130				Tested against F55F8.3 but did not confirm			
let-375	h241				Tested against *imb-1* but did not confirm			
let-387	h87				Tested against *pnk-1* but did not confirm			
let-515	h730				Tested against *rpl-13* but did not confirm			
let-501	h714				Tested against *rpl-4* but did not confirm			
let-361	h97				no candidate			
let-531	h733				no candidate			
let-576	h816				no candidate			
let-518	h316				no candidate			
let-523	h751				no candidate			
let-525	h874				no candidate			
let-584	h743				no candidate			

The asterisk (*) signify a stop codon. Support column describes whether the CDS are lethal when treated with RNAi or a knock-out (K.O.) allele, or both. RNAi Range signifies RNAi lethal phenotype show varying degree of penetrance. Confirmation status notes: ^1^Confirmed by previous publication. ^2^Confirmed by sequencing 2^nd^ allele. ^3^Confirmed by complementation testing. Annotation of human orthologs and associated human conditions are from the literature and public databases such as WormBase and OMIM. The genes are sorted first by confirmation status and then by genomic coordinates.

^a^ *let-103 (h420)* and *let-631 (h502)* have collapsed into *let-363*.

^b^ *let-596 (h782)* and *let-630 (h355)* both confirmed by sequencing a second allele and failed to complement each other. Thus, these two are collapsed into *let-630*.

^c^ *let-104 (h799)* and *let-519 (h405)* have collapsed into *let-526*.

Seven of these genes have been molecularly identified and phenotypically described. *let-603*, an aurora kinase [46], and *let-605*, the cyclin E, had severe gonadal defects [56]. *let-355*, a DEAD box helicase, and *let-384*, an integrator subunit, failed to develop gametes [56]. *let-370*, *let-599*, and *let-604* produced malformed embryos that were not laid or hatched [56]. *let-370* encodes a hexaprenyl pyrophosphate synthetase that is associated with Parkinson’s disease [51]. *let-599* encodes the N-acetyl transferase *nath-10. let-604* encodes *mdt-18*, a mediator subunit. A comprehensive summary of the *let*- encoded products is given in Table 1.

### Novel knock-out alleles provide new genetic resources

We have generated new alleles for 13 genes that currently have no knock-out alleles available: *let-595* (*imb-1*), *let-362 (*Y71G12B.8)*, rnp-6 (let-147), aars-2 (let-366), let-598 (*F27C1.6)*, let-355 (*T05E8.3)*, let-384 (*C06A5.1)*, fars-1 (let-396), let-611 (*C48E7.2)*, mdt-18 (let-604), acdh-5 (let-383), rpb-5 (let-397),* and *let-630 (*Y110A7A.19). Eight of these genes are predicted to have roles in essential basic functions such as transcription or translation. This is not surprising, because we expect genes that function in basic cellular processes to be essential and are best captured using balancer systems. Besides these novel alleles, we have provided additional loss of function alleles for many characterized genes (Table 1). Additional alleles affecting different parts of the gene may disrupt different domains providing an allelic series correlating with different phenotypes.

Genetic strains carrying heritable mutational changes provide a lasting resource that can be used in a variety of experimental conditions and compared to information gained from RNAi knock-down experiments. We cross-checked our high confidence list with the RNAi data annotated in WormBase to see if the lethal phenotype was observed in at least two RNAi experiments. Although for the most part, RNAi data agrees with our mutational data, not every gene was supported by RNAi. We found nine genes showing no lethal phenotype with RNAi and three genes showing lethal phenotype of variable penetrance (Table 1). Of the nine genes that show no RNAi lethal phenotype, six (*inx-12, coq-1, lim-7, tag-146, let-381,* and *let-503*) have additional knock-out alleles that are lethal, suggesting RNAi did not reveal the null phenotype of these genes. The additional information provided by genetic mutation highlights the importance of our collection.

### Essential genes in *sDp2* function in cell cycle and cytokinesis, transcriptional regulation, and RNA processing

**Figure 1:**
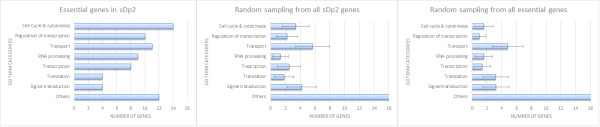
**Functional categorization of essential genes identified in this study using GO terms.** The Y-axis indicates the GO term categories. The X-axis represents the number of genes in each category. Random sampling of 1000 iterations was done by selecting equal number of genes from either all *sDp2* genes or the set of all essential genes identified by RNAi. Error bars represent standard error.

To identify the processes that are essential, we investigated the function of our high confidence gene set along with their orthologs in *D. melanogaster* (fly), *S. cerevisiae* (yeast), and *H. sapiens* (humans). Essential genes are often conserved due to their important biological roles. Fifty-four of our identified essential genes have readily identifiable orthologs in humans [57] (Table 1). We further categorized each gene into at least one of eight functional groups based on their GO annotations (Figure 1). To have a better picture of the roles of different essential genes, multi-functional genes were categorized into more than one functional group. The cell cycle & cytokinesis, transcriptional regulation, transport, RNA processing, and transcription categories contained more genes than did the groups representing translation, signal transduction, and the other groups that includes metabolic and structural processes.

Of these eight functional groups, we found three groups that were significantly enriched in the *sDp2* region when compared to the non-essential genes in *sDp2*: cell cycle & cytokinesis (p = 3.61e^-9^, χ^2^ test), regulation of transcription (p = 6.21e^-8^, χ^2^ test), and RNA processing (p = 6.35e^-12^, χ^2^ test). Our analysis indicates that members of these processes are enriched in essential genes. We have previously shown that components of the spindle assembly checkpoint are essential for survival [58]. Here we showed that genes in the *sDp2* region function in various phases of the cell cycle. For instance, *let-380* (*knl-2)* is critical for loading *hcp-3* (CENP-A) to chromatin and forming the kinetochore [59]. *let-603* (*air-2), let-597 (hcp-4),* and *let-106 (hcp-6)* remove cohesions for proper resolution of centromeric connections and segregation of homologous chromosomes during meiosis [60–62]. *let-365* (*sep-1*) is essential for chromatid separation and proper anaphase. In addition, *let-364* (*mat-1)*, a member of the anaphase promoting complex (APC), is crucial for the transition from metaphase to anaphase [63]*. lin-6* (*mcm-4*) is required for DNA replication and activates a checkpoint when entering into M phase [39]. *let-599* (*nath-10*) and *let-354* (*dhc-1*) are crucial for cytokinesis during cell division [64, 65]. *let-385* (*teg-4*) is a component of splicing complex A that functions in the meiosis entry decision [66, 67]. Our data indicate that disrupting any phase of the cell cycle process can lead to lethality.

Are functions of the essential genes identified in this study representative of all essential genes? Random sampling simulation from 3500 essential genes indicated by RNAi shows a very different GO term distribution (Figure 1). In the larger set samples, we observed that cell cycle and cytokinesis (p = 1.02e^-22^, χ^2^ test), regulation of transcription (p = 2.48e^-20^, χ^2^ test), and RNA processing (p = 5.43e^-10^, χ^2^ test) are under-represented compared to our sequenced set. Although we acknowledge that comparing lethal mutants to RNAi phenocopies is not fully equivalent, at the present time there is not a large enough mutant essential gene collection to do this comparison. It is intriquing nevertheless to raise the question of regional differences in essential gene functions and we look forward to having a more complete dataset that can be used to address this issue.

### Essential gene transcripts are supplied maternally

From the set of 59 essential genes, 34 of them arrest development as embryos or early larvae, indicating that they are important early in development. To test this hypothesis, we analyzed the temporal expression of these genes using RNA-seq divided into 23 separate 30-minute embryonic stages, 4 larval stages, pre-gravid young adult stage, and the young adult stage. The normalized RNA-seq data was obtained from the modENCODE project [68, 69].

**Figure 2:**
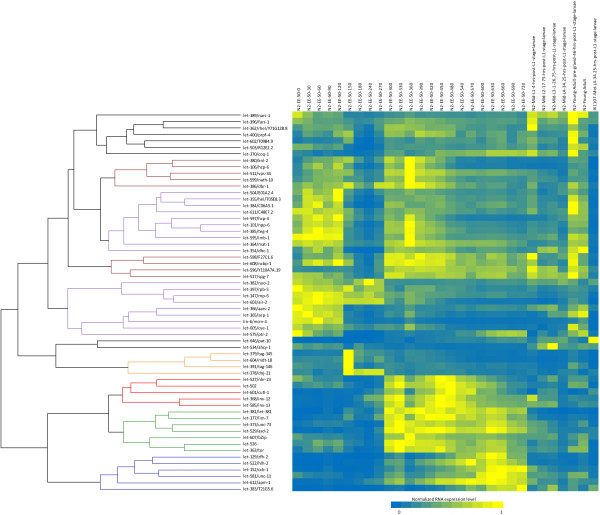
**This figure represents the normalized transcript level (read number per coding length per million reads) for each gene across the developmental stages including 23 embryo stages separated by 30 minute interval, four larval stages (L1-L4), pre-gravid young adult, and gravid young adult.** For comparing germline expression, we’ve included the transcript level from JK1107 carrying a mutation in *glp-1*, which is essential for mitotic germ cell proliferation [71]. The heatmap represents normalized transcript level from high (yellow) to low (blue). Seven distinct clusters that are based on their expression pattern are shown by colored branches. Purple: early-embryonic; Brown: early- and mid-embryonic; Red: mid-embryonic; Blue: late-embryonic; Green: mid- and late-embryonic; Orange: gastrulation; Black: larval.

Seven distinct patterns were seen from the heatmap (Figure 2). Five genes (colored red) express highly during mid-embryonic stage (300 min – 600 min), six genes (colored blue) express highly during late-embryonic stage (600 min – hatch), and seven genes (colored green) express highly in both mid-embryonic and late-embryonic stages. Eighteen genes (colored purple) show elevated expression very early in embryonic development (0 min – 300 min). Most of these genes, however, had a dramatic drop in expression level at 150 min, which is when gastrulation occurs [70]. Observing that many of these genes also show strong expression in young adults but not in larval stages suggests that these messages are highly transcribed in the germline and are likely maternally derived in the embryo. On the other hand, nine genes (colored brown) show some early embryonic expression but have their strongest expression during mid-embryonic stages. A group of four genes (colored orange) show specific expression during gastrulation. Lastly, eight genes (colored black) have elevated expression during specific larval stages.

From the RNAseq data, we observed 18 genes with expression patterns that indicated maternal contribution during early embryogenesis. This ratio is not significantly different from the set of all essential genes. However, when compared with the set of non-essential genes, our essential gene list is significantly enriched for genes with strong maternal contribution (1.24e^-5^, χ^2^ test). These data indicate that many essential genes important for early embryonic development have maternal contribution.

## Conclusions

The function of essential genes is poorly understood. Having a combination of genetic strains for which the molecular identity is known would provide a powerful resource for their study. However, even in the model system *C. elegans*, only about 25% of the essential genes have a knockout alleles. RNAi has also been used to identify essential genes [72, 73]. Despite the success of these studies, only a small subset (~800 genes) have been profiled phenotypically [72]. We have a large collection of mutant strains, but only now has it been technically feasible to easily identify their corresponding coding regions. Our library currently consists of 1350 lethal mutations maintained by balancers in chromosomes I, III, IV, and V, of which chromosome I is the closest to saturation [19]. Recent whole genome screening experiments using the CRISPR/Cas9 system have opened up the possibility of identifying essential genes using this targeted approach. However, targeted approaches directed towards identifying essential genes in an intact multicellular organism are still limited in terms of recovery and maintenance of lethal mutations and impractical for large scale screens. The relative ease of capturing and maintaining lethal mutations makes balancer systems the method of choice for essential gene studies. However, using random mutagenesis is not possible to achieve 100% saturation (finding all essential genes). Small targets have a smaller chance of being mutated and are likely missed in mutagenesis experiments. Also, finding new essential genes in subsequent screenings becomes more and more difficult because the screens follow (approximately) a Poisson distribution giving diminishing returns. Thus, a combination of targeted and forward mutational approaches is best.

We previously developed a pipeline and applied it to the identification of *let-504*[30]. In the analysis presented here, we applied the pipeline to further analyze 76 essential genes on Chromosome I and produced high confidence identification for 64 genes. Some of the confirmed candidates were found outside the mapped region suggesting that the boundaries of the genetically identified zones can be further refined. We have shown that our approach is much more efficient and cost-effective than the traditional method. Assessments from this study will help us improve our identification pipeline and give us the confidence to apply this technique to the rest of our collection of essential genes.

Our results here provide additional alleles to known genes as well as provide new alleles. The added alleles will be valuable for establishing allelic series that may exhibit different phenotypes. For instance *let-147*/*rnp-6* has 4 alleles each showing a different arrest stage [19], suggesting different protein domains are being disrupted. More importantly, our results provided 13 new alleles in essential genes where no alleles existed. The genetic resources provided with our method will be beneficial to the field of essential gene research.

We have demonstrated here that Let mutants can be used, not only individually to study the gene’s function, but analyzed as a group to better understand the functions a living multi-cellular animal needs for survival. Understanding the function of individual essential genes has applications for medicine. Essential genes in bacteria have been exploited to develop new antimicrobials [5]. An understanding of essential genes can be exploited for new medical uses. For example, the human ortholog of *let-400/prpf-4*, has been found to induce G1/S arrest and may function as a cancer suppressor [55]. Therefore, a resource such as described here for identifying and studying essential genes in model organisms has direct benefit.

We have shown that essential genes in the left half of chromosome I in *C. elegans* function in cell cycle control, transcriptional regulation, and RNA processing. Previous reports studying other genomic regions have shown different gene classes such as those regulated by the GATA transcription factor [74] and the sex-regulated genes [75] are non-randomly distributed in the genome. Thus, we believe the organization of these genes within the genome is also non-random. With our method, it is now possible to generate genetic resources to capture the majority of the essential genes. The study of which will provide us with a global picture of the minimum set of genes and pathways that is needed for the survival of a multi-cellular organism, and their organization in the genome. An increased understanding of the nature of essential genes is relevant not only to our knowledge of the biological survival of the organism but also has the potential for better medical procedures.

## Methods

### Strains

The strains used in this study are listed in Table 1. We have listed all the other available alleles for each *let*- gene in Additional file 2. The strains were grown and maintained on nematode growth medium streaked with E. coli OP50 [76]. The strains used in this study were generated by mutagenizing KR235 [*dpy-5 (e61)*, +, *unc-13 (e450)*/*dpy-5(e61)*, *unc-15(e73)*, +; *sDp2*] with 12 mM EMS [35]. Briefly, the treated gravid wildtypes were individually plated on 5 cm plates and wildtype gravid F1s were also individually plated 5 days later. Their progeny (F2s) were screened for the absence of Dpy-5 Unc-13 individuals (Additional file 1). A single Unc-13 animal was transferred to confirm the existence of a lethal mutation. A balanced lethal would exhibit Unc-13 and developmentally arrested Dpy-5 Unc-13 [35]. All the strains were maintained at 20°C and by selecting Unc animals. Each strain was grown from one hermaphrodite and expanded to 20 2-inch plates. The worms were collected by rinsing the plates with M9 (6 g Na_2_HPO_4_, 3 g KH_2_PO_4_, 5 g NaCl, 0.2 g MgSO_4_ in 1 L of H_2_0). The worms were washed with 12 ml of M9 three times and incubated at room temperature for 2 hours. The final pellet was frozen in -80°C.

### Genomic DNA extraction and sequencing

Genomic DNA was extracted by phenol/chloroform as described previously [30]. Briefly, the worm pellet was lysed in 0.5% SDS and 100ug of Proteinase K in 50°C for two hours. DNA was extracted with phenol/chloroform three times and precipitated with 100% ethanol. 20 ug of RNase A was added to the eluted sample to remove RNA contaminants and this was followed by three more rounds of phenol/chloroform extraction and ethanol precipitation. 10 ug of purified genomic DNA was sequenced at the BC Cancer Agency Genome Sciences Centre using Illumina PET HiSeq technology.

### Mutation identification procedure

Sequencing reads were aligned to the WS200 *C. elegans* genome using BWA [36] under default settings. Duplicated reads were filtered with GATK [77]. Further realignment around indels was also done with GATK. The BAM files were analyzed for SNV and small indels using Varscan [78]. The SNVs or indels returned by Varscan were filtered by 1) mutations in the parental strain KR235 mutation, 2) variant ratio (90% > x > 40%), and 3) genomic location (in coding sequences only). Allelic ratio was calculated as the ratio of mutant allele:reference allele. The effect for each CDS from the accumulate effect of the mutations in the genome was analyzed using Coovar [79]. Mutational landscape analysis was done using SNVs exhibiting G > A or C > T transitions as described previously [30]. Each genes in the *sDp2* carrying a non-synonymous mutation was considered and ranked according to the severity of the mutation. Mapping information from [19] was used as a guide to find the most likely mutation. The mutations for each strain can be downloaded from http://lethal.mbb.sfu.ca/jschu/essential_genes.

Sequencing of a second allele was done with Sanger sequencing or WGS. PCR primers were designed using Primer3 [80, 81] spaced 250 bp apart with staggered orientation. This allowed sufficient overlap so that each position was covered at least twice. The Sanger reads were aligned to the wildtype transcript sequence using Clustal [82]. The alignments from each Sanger read were merged and analyzed with Bioedit. A mutation was confirmed if it was supported by all the Sanger reads and the sequencing traces show a clear double peak. A prediction was also confirmed when WGS of a second allele has a different mutation in the same gene.

### Confirmation by complementation testing

Allelic combinations were established previously by complementation testing as described in [19] with the following exceptions. In a few cases, candidate SNVs were found for mutations, which were previously described as mapping to separate zones, in a single coding region. In these cases complementation testing was done between mutations predicted to be in the candidate coding region and confirmed that they did form a single complementation group as shown in Additional file 3.

Strains carrying a lethal mutation were selected for complementation testing with other lethal-carrying strains based on the identification of candidate mutations in the same gene. In order to determine allelism, *let-x dpy-5 unc-13/let-x dpy-5 unc-13; sDp2* hermaphrodites were mated to wild-type males. F1 males (*let-x dpy-5 unc-13*/ + + +) were crossed to hermaphrodites carrying a second lethal (*let-y dpy-5 unc-13/let-y dpy-5 unc-13; sDp2*). The diagnostic phenotype indicating complementation in the progeny of the cross was Dpy Unc males and fertile hermaphrodites (*let-x dpy-5 unc-13/let-y dpy-5 unc-13*). A minimum of ten wild-type males on one plate was considered sufficient to conclude that the absence of Dpy Unc animals was not due to poor mating.

### Gene ontology analysis

Orthologs were predicted by a set of programs consisting of Inparanoid [83], OrthoMCL [84], and Ensembl-Compara [85] with methods as previously described [57]. The protein sets used were: *C. elegans* (WS230), *S. cerevisiae* (64-1-1), *D. melanogaster* (r5.46), and *H. sapiens* (GRCh37.66). GO annotation was done using Blast2GO [86]. GO profile comparison was done using all the genes under *sDp2* and all the essential genes as identified by *RNAi* collected from WormBase WS230.

### RNA-seq expression analysis

Normalized RNA-seq data were downloaded from the modEncode website (http://www.modencode.org). The average normalized read count for each CDS was calculated as the total normalized read count of all coding base-pairs divided by the length of CDS. The expression profile clustering was done using agnes clustering in R.

## Electronic supplementary material

Additional file 1: **This figure describes how lethal mutations are balanced with** ***sDp2*** [35]. KR235 is mutagenized with 12 mM EMS. The treated gravid wildtypes were individually plated on 5 cm plates and wildtype gravid F1s were also individually plated 5 days later. Their progeny (F2s) were screened for the absence of Dpy-5 Unc-13 individuals. A single Unc-13 animal was transferred to confirm the existence of a lethal mutation. A balanced lethal would exhibit Unc-13 and developmentally arrested Dpy-5 Unc-13. The asterisk (*) denotes an EMS mutation. In the F1 generation, the mutation could be on either homolog but not both. (PPTX 42 KB)

Additional file 2: **List of genes studied and their associated alleles.** The alleles used for WGS are listed in the 2^nd^ column. The alleles used for confirmation are noted by an asterisk (*). (XLSX 13 KB)

Additional file 3: **Comparison of genomes missing** ***dpy-5*** **and/or** ***unc-13*** **markers.** The average read depth per 10Kbp of coding element is plotted along the length of chromosome I. The x-axis shows the coordinate in 10 K units. The y-axis shows the number of reads. The control genome show 33% more reads in the first 7 Mbp while the genome with missing markers shows a flat distribution. (PPTX 110 KB)

Additional file 4: **Complementation table for** ***let-363 (h98), let-130 (h216), let-130 (h451), let-631 (h502), let-630 (h355), let-596 (h782), let-526 (h185), let-104 (h799),*** **and** ***let-519 (h405).*** (-) indicates two mutations fail to complement and (+) indicates two mutations complement each other. N.D. indicates the particular combination was not done. (DOCX 13 KB)

## Abbreviations

WGS: Whole genome sequencing
SNV: Single nucleotide variation
EMS: Ethyl methanesulfonate
GO: Gene ontology.
