# Supplementary material for: High-throughput capturing and characterization of mutations in essential genes of Caenorhabditis elegans
Source: BMC Genomics. 2014 May 12;15(1):361. doi: 10.1186/1471-2164-15-361 (PMC4039747; doi:10.1186/1471-2164-15-361)
Supplement: Supplementary file 1 — Additional file 1: This figure describes how lethal mutations are balanced with sDp2 [35]. KR235 is mutagenized with 12 mM EMS. The treated gravid wildtypes were individually plated on 5 cm plates and wildtype gravid F1s were also individually plated 5 days later. Their progeny (F2s) were screened for the absence of Dpy-5 Unc-13 individuals. A single Unc-13 animal was transferred to confirm the existence of a lethal mutation. A balanced lethal would exhibit Unc-13 and developmentally arrested Dpy-5 Unc-13. The asterisk (*) denotes an EMS mutation. In the F1 generation, the mutation could be on either homolog but not both. (PPTX 42 KB) [file 12864_2013_6076_MOESM1_ESM.pptx]

## Slide 1
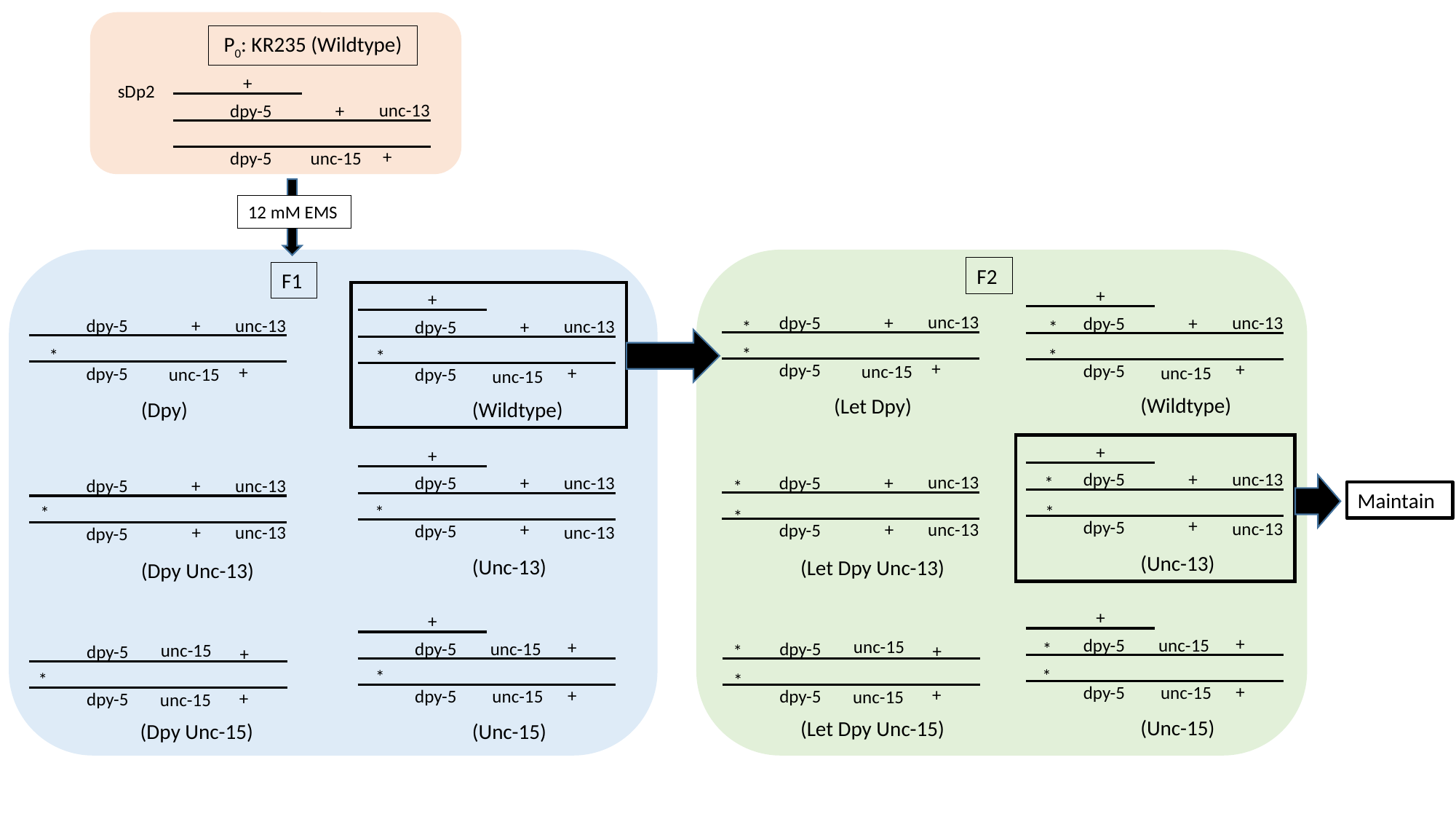

P0: KR235 (Wildtype)
+
unc-13
+
dpy-5
+
unc-15
dpy-5
sDp2
12 mM EMS
F2
F1
+
unc-13
+
dpy-5
+
dpy-5
unc-15
+
unc-13
+
dpy-5
+
dpy-5
unc-15
unc-13
+
dpy-5
+
dpy-5
unc-15
unc-13
+
dpy-5
+
dpy-5
unc-15
*
*
*
*
*
*
(Wildtype)
(Let Dpy)
(Wildtype)
(Dpy)
+
unc-13
+
dpy-5
dpy-5
+
unc-13
+
dpy-5
dpy-5
unc-13
+
dpy-5
dpy-5
*
unc-13
+
dpy-5
dpy-5
*
Maintain
*
*
*
*
+
unc-13
unc-13
+
+
unc-13
unc-13
+
(Unc-13)
(Unc-13)
(Let Dpy Unc-13)
(Dpy Unc-13)
+
dpy-5
+
dpy-5
unc-15
+
dpy-5
+
dpy-5
unc-15
+
unc-15
unc-15
+
dpy-5
+
dpy-5
unc-15
*
unc-15
unc-15
*
+
dpy-5
+
dpy-5
unc-15
+
*
*
*
*
(Unc-15)
(Let Dpy Unc-15)
(Unc-15)
(Dpy Unc-15)
