# Supplementary material for: High-throughput capturing and characterization of mutations in essential genes of Caenorhabditis elegans
Source: BMC Genomics. 2014 May 12;15(1):361. doi: 10.1186/1471-2164-15-361 (PMC4039747; doi:10.1186/1471-2164-15-361)
Supplement: Supplementary file 3 — Additional file 3: Comparison of genomes missing dpy-5 and/or unc-13 markers. The average read depth per 10Kbp of coding element is plotted along the length of chromosome I. The x-axis shows the coordinate in 10 K units. The y-axis shows the number of reads. The control genome show 33% more reads in the first 7 Mbp while the genome with missing markers shows a flat distribution. (PPTX 110 KB) [file 12864_2013_6076_MOESM3_ESM.pptx]

## Slide 1
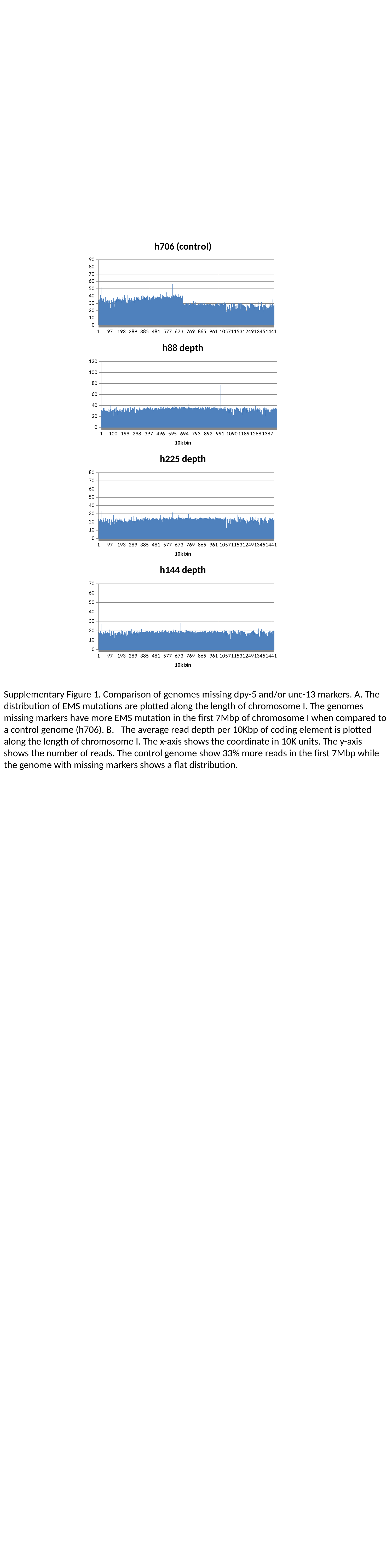

### Chart: h706 (control)
| Category | Average |
|---|---|
### Chart: h88 depth
| Category | |
|---|---|
### Chart: h225 depth
| Category | |
|---|---|
### Chart: h144 depth
| Category | |
|---|---|Supplementary Figure 1. Comparison of genomes missing dpy-5 and/or unc-13 markers. A. The distribution of EMS mutations are plotted along the length of chromosome I. The genomes missing markers have more EMS mutation in the first 7Mbp of chromosome I when compared to a control genome (h706). B. The average read depth per 10Kbp of coding element is plotted along the length of chromosome I. The x-axis shows the coordinate in 10K units. The y-axis shows the number of reads. The control genome show 33% more reads in the first 7Mbp while the genome with missing markers shows a flat distribution.
