# Supplementary material for: High-throughput capturing and characterization of mutations in essential genes of Caenorhabditis elegans
Source: BMC Genomics. 2014 May 12;15(1):361. doi: 10.1186/1471-2164-15-361 (PMC4039747; doi:10.1186/1471-2164-15-361)
Supplement: Supplementary file 4 — Additional file 4: Complementation table for let-363 (h98), let-130 (h216), let-130 (h451), let-631 (h502), let-630 (h355), let-596 (h782), let-526 (h185), let-104 (h799), and let-519 (h405). (-) indicates two mutations fail to complement and (+) indicates two mutations complement each other. N.D. indicates the particular combination was not done. (DOCX 13 KB) [file 12864_2013_6076_MOESM4_ESM.docx]

Supplementary Table 1. Complementation table for *let-363 (h98), let-130 (h216), let-130 (h451), let-631 (h502), let-630 (h355), let-596 (h782), let-526 (h185), let-104 (h799),* and *let-519 (h405).* (-) indicates two genes fail to complement and (+) indicates two genes complement each other. N.D. indicates the particular combination was not done.

|  | *h98* | *h216* | *h451* | *h502* | *h355* | *h782* | *h185* | *h799* | *h405* |
| --- | --- | --- | --- | --- | --- | --- | --- | --- | --- |
| *let-363 (h98)* | **-** | **-** | **-** | **-** | **+** | **+** | **+** | **+** | **+** |
| *let-130 (h216)* |  | **-** | **-** | **-** | n.d. | n.d. | **+** | **+** | **+** |
| *let-130 (h451)* |  |  | **-** | **-** | n.d. | n.d. | **+** | **+** | **+** |
| *let-631 (h502)* |  |  |  | **-** | n.d. | n.d. | **+** | **+** | **+** |
| *let-630 (h355)* |  |  |  |  | **-** | **-** | n.d. | n.d. | n.d. |
| *let-596 (h782)* |  |  |  |  |  | **-** | n.d. | n.d. | n.d. |
| *let-526 (h185)* |  |  |  |  |  |  | **-** | **-** | **-** |
| *let-104 (h799)* |  |  |  |  |  |  |  | **-** | **-** |
| *let-519 (h405)* |  |  |  |  |  |  |  |  | **-** |
